# Editorialets

**Published:** 1909-10

**Authors:** 


### Editorialets.

Hurrah for Cook! Peary is a cad.

---

Dr. J. Hicks Florence, Quarantine Officer at Galveston, has been chosen Medical Director of the Great Southern Life Insurance Company and will remove to Houston.

AND Harriman didn't leave a cent for religion, charity, education or science.

---

BEWARE of flannels. Wear cotton or linen next to the skin.

---

DR. GEO. H. HARWOOD has removed from Marble Falls to Sabinal.

---

DR. R. L. GRAHAM has removed from Skidmore to Harlingen.

---

WANTED.—A superintendent at a salary of $3000. Apply to Governor Campbell. The Legislature appropriated $40,000 for a "leprosarium" and a superintendent with $3000 salary. To date not an applicant has appeared.

---

ALL BUT TEXAS.—Twenty-eight States have appropriated an aggregate of $4,000,000 to prevent the spread of tuberculosis. Our most grave and reverend Governor vetoed the bill for a tuberculosis sanitarium, notwithstanding that the medical profession unanimously asked for it; both branches of the Legislature asked for it unanimously; the Elks, the Woodmen, the Travelers' Protective Association, and every other fraternal order begged for it. By this act of shortsighted wisdom in disregarding the wishes of the people in their prayer for protection he has disappointed and alienated hosts of his friends and once staunch supporters.

---

LISTERINE is an ideal intestinal antiseptic, and finds its best field in typhoid, diarrhea, dysentery, indigestion, etc. It is pleasant to take and harmless. It will arrest fermentation and cut short an indigestion, relieve nausea and "looseness of the bowels." Moreover, it is an admirable toilet article, used as gargle or mouth wash, and is almost a specific for prickly heat, mosquito bites, etc. I know whereof I speak.

---

THE TEXAS STATE BOARD OF HEALTH has perfected the mandatory portion of the sanitary code provided for by the bill, and will soon promulgate it. The "advisory" portion will be acted on by a convention of county health officers to meet at Austin October 6, 7 and 8 inst. A large attendance is expected.

Pellagra means "rough skin." It is supposed to be caused by diseased corn, just as beri-beri is caused by diseased rice. "Smut" in corn is a fungus similar to that of rye, "spurred rye," from which we get ergot. Write to Dr. Lavinder, Marine Hospital Service, or the Surgeon-General, for a copy of Surgeon Lavinder's write-up of the subject.

---

New York, September 11, 1909.

*F. E. Daniel, Editor Texas Medical Journal, Austin, Texas.*

Dear Doctor: The writer has this day purchased "Pediatrics." Perhaps you will recall that this journal was established in 1896 by Dr. Dillon Brown, of New York, and since its foundation it has been the leading exponent of pediatric teaching in this country. It is the editor's aim and intention to make it the greatest and most helpful journal in the teaching of diseases of children in English or any other language. The first issue under the new management will appear October 10th.

Very sincerely yours,
W. E. Fitch, M. D.,
Editor and Publisher Gaillard's Southern Medicine, N. Y.

---

A Sanitarium for Alcoholic and Drug Patients.—Dr. Givens' sanitarium for nervous and mental diseases at Stamford, Conn., has a separate department for alcoholic and drug patients, and the statute of Connecticut permits such patients to voluntarily commit themselves for a period not exceeding one year. The regular, systematic life under medical supervision is excellent. Write Dr. Givens, Stamford, Conn., for particulars.

---

Barnes University Appointments.—The trustees of the Barnes University of St. Louis have recently increased the teaching staff of the Medical Department. Dr. James Moores Ball has accepted the Chair of Ophthalmology and Ophthalmic Surgery. Dr. J. J. Houwink has been elected Professor and Head of the Department of Dermatology and Syphilology. Additional laboratory equipment has been installed and the medical buildings have been thoroughly renovated in preparation for the winter's work. The Centenary Hospital, with 100 beds, adjoins the medical school and is a valuable asset for clinical teaching.

Barnes University,
J. K. Goin, President.

MEDICAL JOURNALS.—Any physician and surgeon with four or six of our modern leading one-dollar journals should be a first-class fan if he can read and understands what he reads. I am taking one or two journals that my father took forty years ago. I must have my medical journals. I am taking five independent journals. I figure I can get more out of five one-dollar journals than out of a five-dollar association organ. I would not trade any of my journals for an association organ. You ask why this is? Because the independent editors are free as the air we breathe; they give us straight goods; they are real helpers. And second, the independent journals are not filled with association news. Association business will not save a patient's life. Draw your own conclusions.—Frank L. Fuller, M. D., in *Medical Standard.*

---

DAVID FINNEY STUART, M. D., Jefferson Medical College, Philadelphia, 1859; chief surgeon of the Houston and Texas Central and Houston East and West Texas railroads from 1878-1895; president of the Houston Infirmary Sanitarium; formerly president of the board of trustees of Texas Medical College; assistant surgeon of the Tenth Texas Infantry and senior surgeon of Granberry's Texas Brigade in the Civil War; in 1871 vice-president and two years later president of the State Medical Association of Texas; died at his home in Houston, September 8, aged 76.

---

DR. F. C. FORD, quarantine officer at Sabine Pass, succeeds Dr. J. Hicks Florence as quarantine officer at Galveston, Dr. Florence having resigned and is now Medical Director Southern Life Insurance Co. Office at Houston.

---

THE article on "Pellagra" on editorial page was contributed by the Marine Hospital Service and Public Health Department at Washington to the medical press generally to disseminate the information and insure a wider distribution of Dr. Lavinder's pamphlet. It will appear or has appeared in other journals.

---

DON'T fail to attend the convention of the great Medical Association of the Southwest at San Antonio, November 9th, 10th and 11th. The Fair will be in full swing at that time. Special rates, and we may expect a royal time. Build up this great association and amputate it from the Octopus.

THE "RED BACK" IN INDIA.

---

THE PRACTICAL MEDICINE,

A Monthly Medical Journal for the Busy Practitioners.

ADVERTISING DEPARTMENT.

DELHI, INDIA, May 8, 1909.

*To the Publishers Texas Medical Journal, Austin.*

GENTLEMEN: We are desirous of inserting the enclosed advertisement of our Directory in your paper and would be obliged by your kindly sending us a specimen copy together with your lowest contract rates for special positions for periods of 3, 6 and 12 months, respectively.

On receipt of specimen copy and the information above mentioned, we will be pleased to go further into this matter, in the meantime thanking you in anticipation for the courtesy of a prompt reply.

We remain,

Yours faithfully,

PURENESBRE DOS,

Advertising Manager "Practical Medicine."

THE

**Medical Directory**

OF

**India, Burma & Ceylon**

**SECOND EDITION**

**1909.**

**Price per copy:—**

1£ 10s. ($7.50) ordinary binding.
2£ ($10.00) superior binding and interleaved.

**Sent all charges prepaid on receipt of price.**

Address:—

**'PRACTICAL MEDICINE'**

**Delhi (India).**

London Agents:—W. & G. FOYLE,
135, Charing Cross Road, W. C.

A New Charter.—"A reliable gentleman just from the front" informs us that the Trustees of the A. M. A. will apply for a charter changing the name to the American Mixed Medical Association (Simmons & Co., Ltd.).

---

Operation for cancer of the stomach after the diagnosis has been made by the presence of a palpable tumor can not be hoped to be curative. The hopeful cases are those in which diagnosis is made through an exploratory opening which may be made under cocaine and only large enough to admit the finger.—*American Journal of Surgery.*

